# The Memory section mission

**DOI:** 10.3389/fcogn.2024.1415643

**Published:** 2024-05-07

**Authors:** Marian E. Berryhill

**Affiliations:** Program in Cognitive and Brain Sciences, Program in Integrative Neuroscience, Department of Psychology, University of Nevada, Reno, Reno, NV, United States

**Keywords:** memory, semantic memory, procedural memory, working memory, cognitive psychology, cognitive neuroscience, interdisciplinarity

Frontiers in Cognition is an open access journal currently lead by Field Chief Editor George R. Mangun (UC Davis). Frontiers in Cognition aims to cover the full breadth of basic research in cognition, including major areas such as sensation, attention, decision making, motor planning, etc. In addition, to cognitive psychology and cognitive neuroscience, it welcomes findings from related fields that can inform human cognition, including computational modeling and comparative species research. An additional emphasis is that there is encouragement for contributions that will contribute to “good health and well-being”, which is one of the UN's Sustainable Development Goals. In other words, the Journal explicitly holds the aspirational goal of understanding cognition as well as improving the quality of life of our fellow humans. The Journal is structured to include several Specialty Sections. The purpose of this editorial piece is to provide something of an overarching framework and to define some territory for the Specialty Section entitled: *Memory*.

“Memory” is a general term referring to the fact that some aspect of a prior experience influences current behavior and/or cognition. The term “memory” may not particularly meaningful to the scientific community when speaking amongst themselves because it covers truly vast territory. However, in the Memory section, we would prefer it be considered as an open invitation to include the broadest array of memory researchers to submit. A primary goal is to provide a broad, deep forum for research findings across all memory subfields. There is great value in serving as a forum for memory and as a platform for dissemination to the largest population possible. A criticism of this “big tent” embrace would be that we fail to provide useful integration of current advances in memory. We will work to counter this potential danger by encouraging Special Topics to tap exciting current issues in a deeper, more focused manner. Special Topics are collections of papers addressing a more narrowly defined topic of interest. These Special Topics are highly democratic, and if you have an idea for one, please contact any of the editorial staff to discuss your idea, or follow the instructions in the Journal Special Topics page.

A second goal of the Memory section is to encourage collaboration and integration across memory subfields. In other words, it is all too easy to dig deeper in a niche area without recognizing you are in a deep well. We encourage labs to collaborate across different memory subfields to build stronger science. Collaborations across levels of neuroscience scale are also welcome. By this, we mean, we welcome research that can provide insights regarding human memory by incorporating findings from disparate fields including (but not limited to) cellular/molecular neuroscience, clinical fields (clinical psychology, neuropsychology, neurology, occupational therapy, etc.), genetics, and a host of -*omics* (e.g., proteomics). For there to be a complete accounting of memory function there will ultimately need to be a major push toward interdisciplinary integration across all levels of scale. This will cover territory currently within most fields of science, including chemistry, physics, physiology, psychology, biology, and neuroscience, among others. Admittedly, the horizon on this goal is not yet visible, but that does not mean we cannot make progress in this direction. Through innovative collaborations we will better understand intact and pathological memory, and be pushed to address the assumptions of our individual fields.

We also extend the aims of the Journal to expressly embrace the “good health and well-being” mission by noting that this mission extends to the research teams as well as to the implications of the findings. Collaborations within and across subfields can strengthen research teams and build community. One of the lasting lessons of the pandemic is that humans are social beings (more or less), and we suffer when we cannot be engaged in meaningful pursuits with each other. People coming together to work on a research project of mutual interest is one of the great pleasures of the academic life. We encourage growth of collaborations to improve the science and improve the human connections.

On a related note, there is an important and growing role for *adversarial* collaborations between groups advocating for different interpretations of the data. The expansion of empirical methods over the last few decades initiated an extended period of bold and innovative empiricism. Furthermore, we are in a period of revisiting major findings and working to replicate, reframe or reject them. Science is a process that involves refinement. It is now time for research teams to assemble converging findings to challenge and further develop the synthetic theory to accommodate the plethora of new findings and to inspire new perspectives. Thus, adversarial collaborations are well-timed to design and test the key findings that will enable adjudication between competing theoretical perspectives. The *Memory* section offers a home for all of these types of contributions.

The overall goal of the Memory section is to provide researchers with new findings and an outlet for their data, updates on theory, encouragement for innovative collaboration. Memory is a fundamental cognitive attribute. We must advance our understanding of the basic science to for deeper understanding and to improve translational/applied outcomes. Together, we look forward to a long future of contemplating and advancing Memory research.

## Author contributions

MB: Writing – original draft, Writing – review & editing.

